# Benchmark Study of 2D and 3D Gait Analysis for AI-Powered Mobile Fall Risk Screeners

**DOI:** 10.1093/geroni/igaf122.4076

**Published:** 2025-12-31

**Authors:** Zengyan Wang, Chen Chen, Chitra Banarjee, Rui Xie, Ladda Thiamwong

**Affiliations:** University of Central Florida, Orlando, Florida, United States; University of Central Florida, Orlando, Florida, United States; University of Central Florida, Orlando, Florida, United States; University of Central Florida, Orlando, Florida, United States; University of Central Florida, Orlando, Florida, United States

## Abstract

Mobile health clinics are commonly used to assess health but rarely equipped to assess fall risk indicators such as gait impairment. To address this gap, we propose AI-driven mobile units integrating video-based gait analysis and inertial measurement units (IMUs) for rapid, low-cost fall risk screening. This study benchmarked eight 2D and 3D gait extraction models (OpenPose, AlphaPose, HRNet, MediaPipe, ViTPose, videopose, MotionBERT, MotionAGFormer) using publicly available videos of older adults completing the Timed Up and Go test (N = 28), which includes one front-facing camera view and another with a higher, top-down viewing angle. Models were compared in their computational, temporal, and spatial gait metrics (including gait speed, cadence, stride time and length). Results show that 2D methods, particularly HRNet, consistently outperformed other models in both successful recognition rate (HRNet 99% vs 88.3% averaged across all other models). HRNet delivered the most reliable ankle tracking and the fastest inference time, key advantages for resource-constrained environments like mobile units. By contrast, 3D models required more computational resources without improving gait recognition. Temporal metrics from the videos were comparable to IMU-derived metrics. However, Spatial alignment with IMU-derived pelvis trajectories failed across all models. While the 2-camera setup provides stable perspectives for gait analyses, it limits visibility of certain side-plane movements, reducing confidence in spatial parameters such as ankle flexion and knee angle. The superior performance of 2D models confirms the feasibility of deploying lightweight, AI-powered mobile systems for fall risk screening, especially in underserved areas with limited access to specialized geriatric care.

